# Using a stacked-autoencoder neural network model to estimate sea state bias for a radar altimeter

**DOI:** 10.1371/journal.pone.0208989

**Published:** 2018-12-17

**Authors:** Xiangying Miao, Hongli Miao, Yongjun Jia, Yingting Guo

**Affiliations:** 1 College of Information Science and Engineering, Ocean University of China, Qingdao, Shandong, China; 2 National Satellite Ocean Application Service, State Oceanic Administration, Beijing, China; Newcastle University, UNITED KINGDOM

## Abstract

This paper constructed a stacked-autoencoder neural network model (SAE model) to estimate sea state bias (SSB) based on radar altimeter data. Six cycles of the geophysical data record (GDR) from Jason-1/2 radar altimeters were used as a training dataset, and the other 2 cycles of the GDR from Jason-1/2 were used for testing. The inputs to this SAE model include the significant wave height (SWH), wind speed (U), sea surface height (SSH), backscatter coefficient (σ_0_) and automatic gain control (AGC), and the model outputs the SSB. The model includes one input layer, three hidden layers and one output layer. The SSBs in the GDR of Jason-1/2 were obtained from a nonparametric model based on the SWH and U as input variables; thus, the model has high accuracy but low efficiency. The SSBs in the GDR of HY-2A were computed using a four-parameter parametric model that uses the SWH and U as input variables; therefore, this model’s computational speed is high but its accuracy is low. Thus, we used the HY-2A radar altimeter as an unseen validation dataset to evaluate the performance of the SAE model. Then, we analyzed the contrasting results of these methods, including the differences in the SSB, explained variance, residual error and operational efficiency. The results demonstrate not only that the accuracy of the SAE model is superior to that of the conventional parametric model but also that its operational efficiency is better than that of the nonparametric model.

## Introduction

Satellite radar altimeters can quickly measure the global sea surface height (SSH) and invert geophysical information, such as the significant wave height (SWH) and wind speed (U) [1]. With the development of precision orbit determination (POD) technology, sea state bias (SSB) measurements surpass the orbit error and become the most important part of the height measurement errors. Therefore, accurate estimation of SSB can greatly improve the accuracy of height measurements obtained via a radar altimeter. Generally, two ways exist to estimate SSB: theoretical models and empirical models. However, theoretical models are essentially impractical because obtaining a suitable function and the necessary parameters is difficult [2]. The commonly used empirical models include parametric models and nonparametric models. Parametric models are suitable for real-time data processing due to their reasonably extensibility and high computational efficiency. The least squares method is used to determine the coefficient of the functional formula with assumptions of the overall data distribution and functional forms. However, the accuracy of the SSB is limited because the parametric models use the modeling value obtained by the mismatch in the SSH at the cross point or collinearity data, and the assumed functional formula may be incorrect [3–5]. In contrast, nonparametric models obtain the required information from the data itself rather than assuming the overall data distribution and functional form. Nonparametric models can describe the subtle changes of the curve well by fitting the curve between the function and the variable using the exact least squares method. The altimeter Jason-1/2/3 series use a nonparametric model to estimate the SSB and exhibits substantial improvements in mid- and high-latitude areas. The Jason-1/2/3 nonparametric model is built on a kernel-smoothing statistical technique. However, the modeling process is complicated and requires large amounts of computation, which causes low efficiency and inferior extensibility [6–8].

A stacked-autoencoder neural network model (SAE model) is an unsupervised learning network composed of multiple layers of sparse autoencoders [9–10]. A layer-by-layer greedy training method was utilized in the SAE model [11–12] and compared with other neural network models such as back propagation (BP). Based on Bengio's theory, Vincent proposed a denoising autoencoder algorithm (improved AE) that effectively solved the problem of negative results when the distribution has large differences between training samples and test samples [13].

In this paper, the true value of the SSB based on the results of the SSB nonparametric model in the Jason1/2 altimeter geophysical data record (GDR) is used during model training. The input variables, which are the SWH, wind speed (U), SSH, automatic gain control (AGC) and backscatter coefficient (σ_0_), were successively added to the model. Finally, the optimal model was determined via testing. We then used the HY-2A altimeter data as the unseen validation data to evaluate the model’s validity. For all models, Ku-band data from the GDR data set was used for training, testing and application.

## Model training and testing

### Dataset

In this paper, 6 cycles of the GDR data (2,587,994 groups) from the Jason-1 and Jason-2 radar altimeters collected in February, June and October 2004 and 2015 (the 77^th^, 89^th^ and 101^st^ cycles of Jason-1 and the 243^rd^, 255^th^, 267^th^ cycles of Jason-2) were combined as a training set. In addition, 2 cycles of the GDR data (the 60^th^ cycle of Jason-1 and the 158^th^ cycle of Jason-2, 831,642 groups) were used for testing.

Based on the quality requirements of the radar altimeter data and SSB, error correction values, such as instrument errors, dry and wet troposphere delays, ionosphere delays and inverse atmospheric pressure, high frequency oscillations, tides, pole tides, solid earth tides and load tides are included in the altimeter height measurement data [7,8]. Abnormal data (SSH>100 m or SSH<-130 m, SWH<0 m or SWH>11 m, U<0 m/s or U>21 m/s, σ_0_<7 dB or σ_0_>30 dB and AGC>30 dB or AGC<7 dB) were removed, and the data set was normalized. The SWH, U, SSH, σ_*0*_ and AGC values in the GDR were used as the model inputs, while the SSB in the GDR was used as the desired model output when training the neural network.

### Model training

Artificial neural network models are composed of one input layer, one or more hidden layers and an output layer. Generally, the numbers of layers and neurons are not fixed; instead, they are determined by empirical methods depending on the complexity of the problem. When there are too many layers and neurons it may require excessive time to learn the samples; in contrast, when there are too few layers, the fault-tolerance and sample identification performance will fall to a low level [14]. The number of neurons in each hidden layer is typically set to (2, 4, 2) when there are 3 hidden layers and when input neurons have two variables.

**Fig 1:**
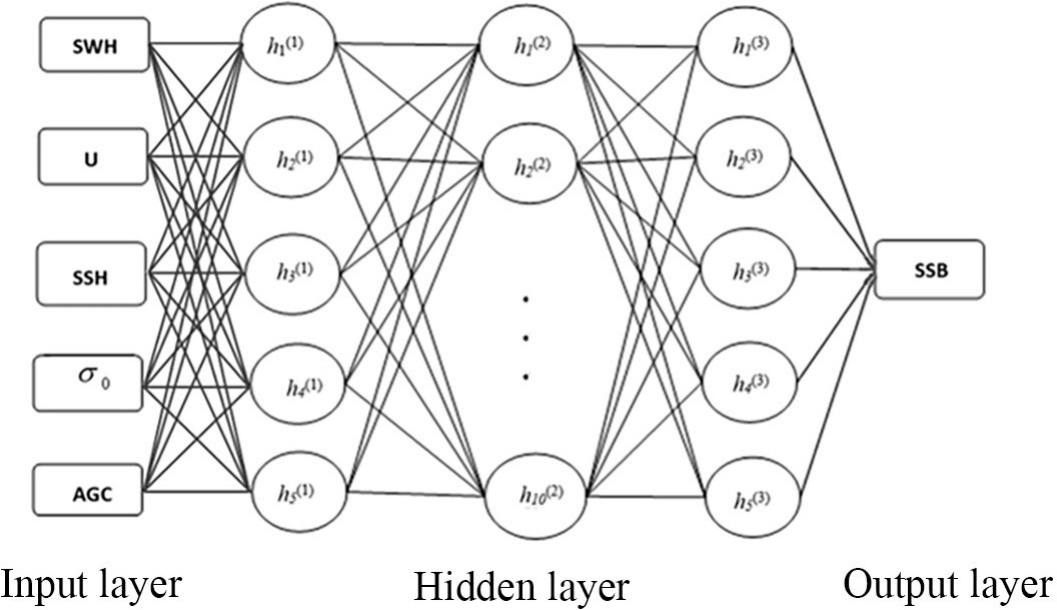
Stacked-autoencoder neural network model.

In our study, the input layer components are the SWH, U, SSH, AGC and σ_0_. The output layer has one element: SSB. We performed a series of data testing, using the same dataset by changing the number of hidden layers and the number of neurons in each layer. The results showed that the symmetrical 3 hidden layer structure has the best effect when the number of neurons in the first hidden layer is equal to the number of inputs. Therefore, the SAE model has three hidden layers structured as (5, 10, 5), as shown in Fig 1.

During forward propagation, any weighted input $z_{j}^{l}$ of the neurons *j* in layer *l* is computed by the activations of the upper layer $a_{j}^{\left(l-1\right)}$ with the weight $W_{jk}^{l}$ between the adjacent layers and the bias $b_{j}^{l}$ of the current layer [15]. Then, a sigmoid activation function *f*(*z*) is used to compute the activations of the current layer $a_{j}^{l}$:

$$z_{j}^{l}=\sum_{k}W_{jk}^{l}a_{k}^{\left(l-1\right)}+b_{j}^{l}$$ (1)

$$a_{j}^{l}=f(z_{j}^{l})$$ (2)

$$f\left(z\right)=\frac{1}{1+e^{-z}}$$ (3)

where *l* is the number of hidden layers (*l*∈[1,3]), *j* is the number of neurons in the current layer, and *k* is the number of neurons in the upper layer. When *l* is equal to 0, the input layer values and the values of $a_{j}^{0}$ are specified by the user. The activations of output layer *d*^*L*^ are the values of the output neurons. *L* is equal to 4 when the quantity of hidden layers is 3 using Eqs (1)–(3).

The main goal of backpropagation in a neural network is to obtain the expressions for the partial derivatives ∂*C*/∂*W* and ∂*C*/∂*b* of the cost function *C* with respect to any weight (*W*) and bias (*b*). During this process, the neural network will adjust the weight and bias values depending on the error between the desired output and the model output until the error falls below a set threshold. The quadratic cost function has the following form:

$$C=\frac{1}{2N}\sum_{i}{\left({\overset{⌢}{y}}_{i}-y_{i}\right)}^{2},$$ (4)

where *N* is the total number of training examples, *ŷ* is the desired output obtained by the GDR, and *y* is the model output from the neural network.

In the output layer, the error components *δ*^*L*^ are given by

$$\delta_{j}^{L}=\frac{\partial C}{\partial a_{j}^{L}}f^{\prime }\left(z_{j}^{L}\right).$$ (5)

The first term on the right, $\partial C/\partial a_{j}^{L}$, measures how fast the cost function is changing at $a_{j}^{L}$, while the second term on the right, $f^{\prime }\left(z_{j}^{L}\right)$, measures how fast the activation function is changing at $z_{j}^{L}$.

In any hidden layer, the error *δ*^*l*^ needs to be computed from the error in the subsequent layer *δ*^*l*+1^ as follows:

$$\delta_{j}^{l}=\left({\left(W_{j}^{l+1}\right)}^{T}\delta_{j}^{l+1}\right)*f^{\prime }\left(z_{j}^{l}\right),$$ (6)

where * represents taking the Hadamard product, which is the elementwise product of two vectors, and ${\left(W_{j}^{l+1}\right)}^{T}$ is the transposition of the weight matrix $W_{j}^{l+1}$. Then, we can obtain the partial derivative of the cost function *C* with respect to the weight and bias:

$$\frac{\partial C}{\partial W_{jk}^{l}}=a_{k}^{l-1}\delta_{j}^{l}$$ (7)

$$\frac{\partial C}{\partial b_{j}^{l}}=\delta_{j}^{l}.$$ (8)

In this paper, we constructed an SAE model. During the training phase of this model, SWH, U, SSH, σ_0_ and AGC are considered the inputs to the SAE model and the corresponding SSB is the output. After multiple forward propagations and backpropagations, the error between the desired output and model output will be less than the set threshold, the output layer neurons will reach saturation, the weight learning and bias learning will terminate, and the weights *W* and bias *b* of this model will be confirmed.

### Model testing

The 60^th^ cycle of Jason-1 and the 158^th^ cycle of Jason-2 were selected for model testing. The effectiveness of the model is evaluated via the correlation coefficient (*r*) and the root mean square deviation (RMS) of the model output SSB_SAE_ (SSB calculated by the SAE model) versus the SSB_GDR_ (SSB from the GDR) from the test set. As *r* approaches 1, SSB_SAE_ and SSB_GDR_ become more highly correlated. The difference between SSB_SAE_ and SSB_GDR_ is smaller when the RMS is closer to 0, and the model is more effective. The results obtained on the validation set were *r* = 0.99 and RMS = 0.5 cm. The averaged absolute value of SSB_SAE_ and SSB_GDR_ are 7.35 cm and 7.20 cm, respectively; therefore, the mean variation is 0.15 cm. The model tests showed that SSB_SAE_ and SSB_GDR_ are highly consistent, implying that the SAE model is both effective and stable.

## Model validation

To evaluate the performance of the trained SAE model, the 70^th^ and 71^st^ cycles (798,726 groups) of the HY-2A radar altimeter were used as the unseen validation dataset. Data from these two cycles are of high quality because they were reprocessed by the National Satellite Ocean Application Service of China (NSOAS). A conventional four-parameter model isapplied in the SSB estimation of HY-2A. This parametric model is a biquadratic, multivariable, Taylor polynomial function for SSB that takes SWH and U as inputs. The parameters in this polynomial function are determined by a regression method based on a least squares estimation.

The output value of the SAE model is notated as SSB_SAE,_ and the value of the parameter model for the GDR of the 70^th^ and 71^st^ cycles of HY-2A is notated as SSB_PM_. To further verify the effectiveness of the SAE model, the following analyses of these two models are conducted: the SSB difference analysis, explained variance analysis, residual error analysis and operational efficiency analysis.

### SSB difference analysis

**Fig 2:**
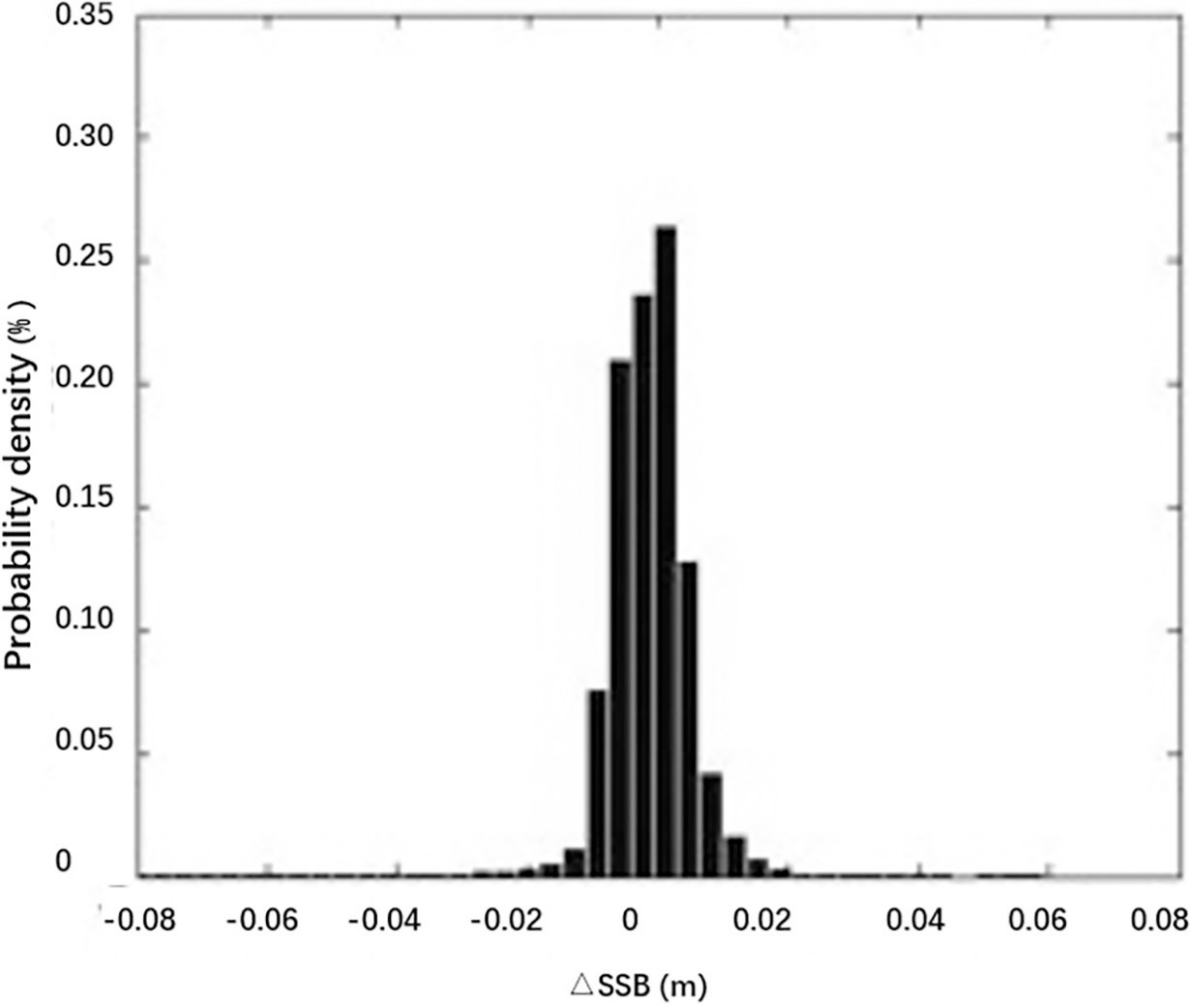
Probability density distribution diagrams of ΔSSB.

**Fig 3:**
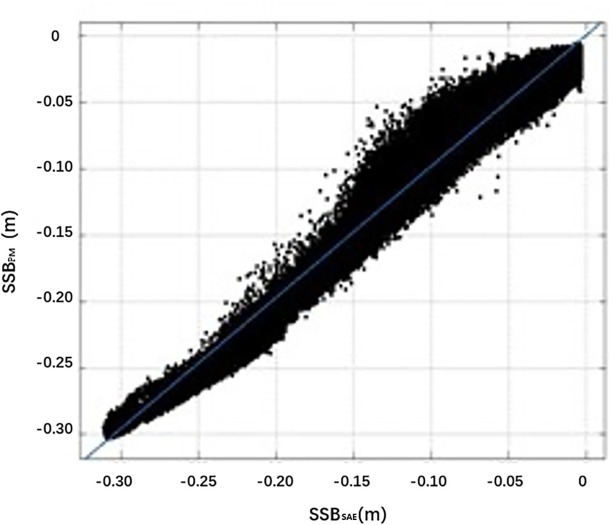
Scatter graph showing the relationship between SSB_SAE_ and SSB_PM_.

The density distribution of the difference ΔSSB between SSB_SAE_ and SSB_PM_ (Δ*SSB* = *SSB*_*SAE*_−*SSB*_*PM*_) is shown in Fig 2, and a scatter diagram with a linear regression line is shown in Fig 3.

The RMS error of the two models is 0.58 cm. The average absolute SSB value of the SAE model is 7.58 cm—an average of 0.57 cm below the average absolute SSB from the HY-2A GDR, which is 8.15 cm. Fig 2 shows that the majority of the differences are concentrated in the range from -1 cm to 1 cm. The graph shows positive skewness because the average SSB bias is negative. Combined with the related scatter plot in Fig 3, the correlation coefficient *r* is 0.96, and, overall, the two values tend to be consistent.

### Explained variance

The explained variance (D) is defined as the difference between the variance in the SSH deviation, **Δ**SSH, at a cross point without SSB correction and the variance in the SSH residual error (*ΔSSH*-*ΔSSB*) at a cross point with SSB correction. The explained variance is the portion of the variance in **Δ**SSH that can be explained by the SSB. This quantity can be used to evaluate the validity and accuracy of the SSB model. Larger values reflect better effectiveness and higher accuracy.

The cross points in the 70^th^ and 71^st^ cycle of the HY-2A radar altimeter (2,997 groups) were extracted. The ascending and descending trajectory SSH values at the cross points were corrected using the SSB results from the SAE model and the parametric model from the GDR of HY-2A. The results show that the explained variance in the SAE model was 31.82 cm^2^ comparedwith 30.69 cm^2^ computed for the explained variance in the parametric model. This result means that the SAE model is more accurate than the conventional parametric model.

### Residual error

The correlation analysis of the SWH, U, σ_0_ and AGC versus the residual error based on the 2,997 groups of cross point data was performed using the results of the two models. The correlation between the average residuals of each segment and the SSH deviation value is not analyzed in this paper because they have a direct relationship.

**Fig 4:**
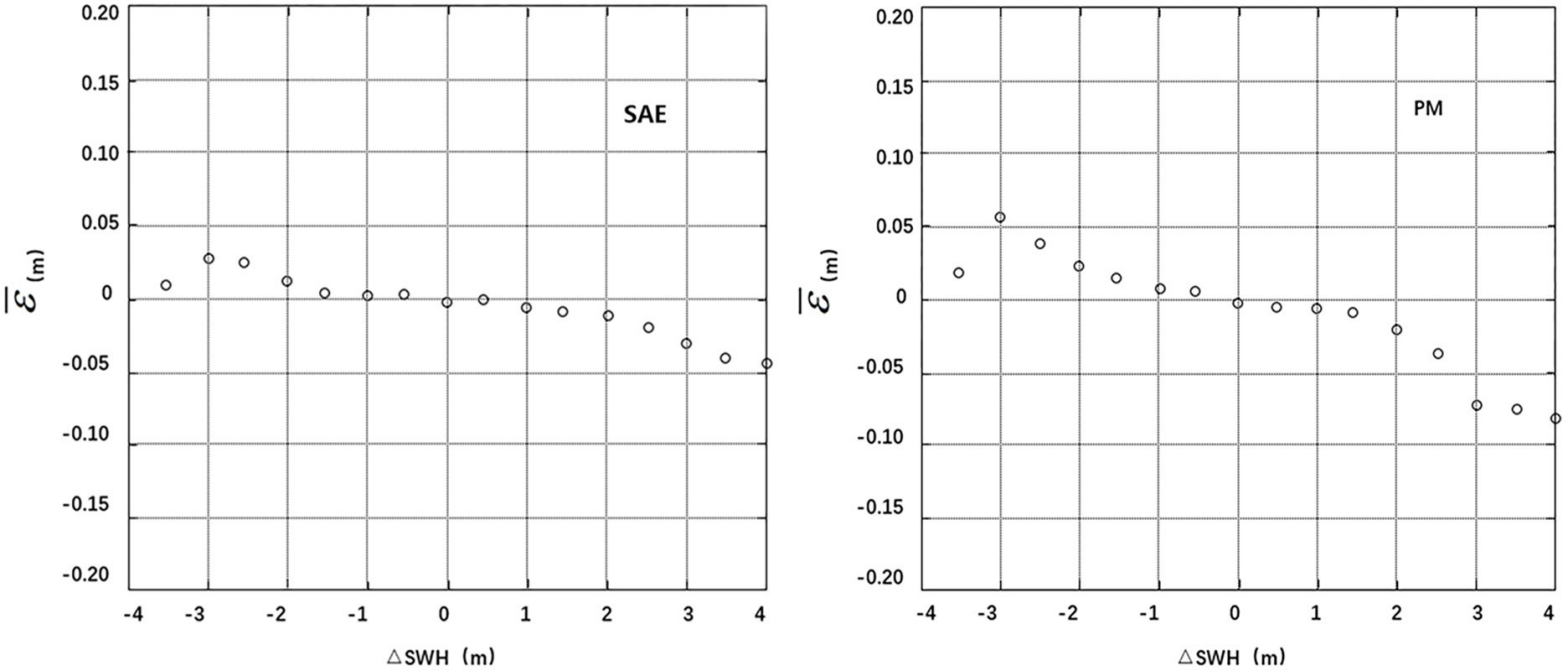
Distributions of mean residuals $\bar{\varepsilon }$ according to ΔSWH.

**Fig 5:**
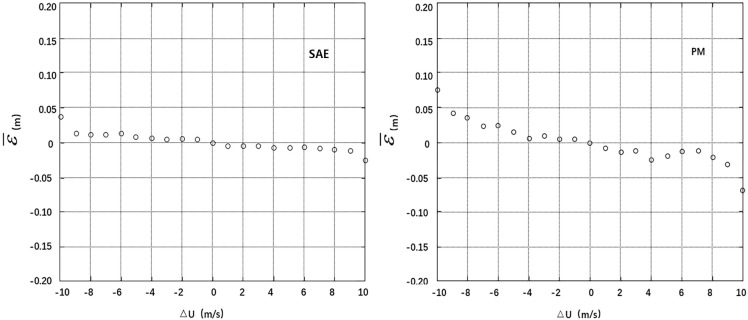
Distributions of mean residuals $\bar{\varepsilon }$ according to ΔU.

**Fig 6:**
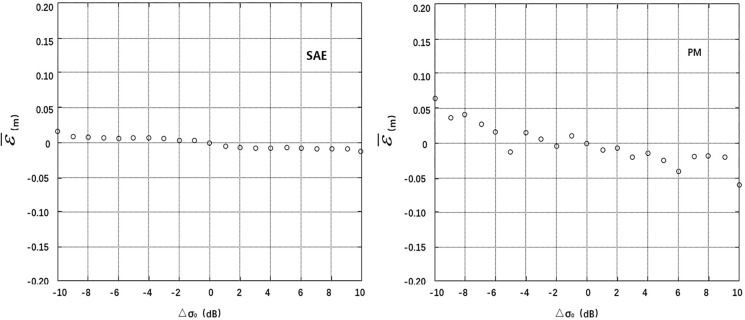
Distributions of mean residuals $\bar{\varepsilon }$ according to Δσ_0_.

**Fig 7:**
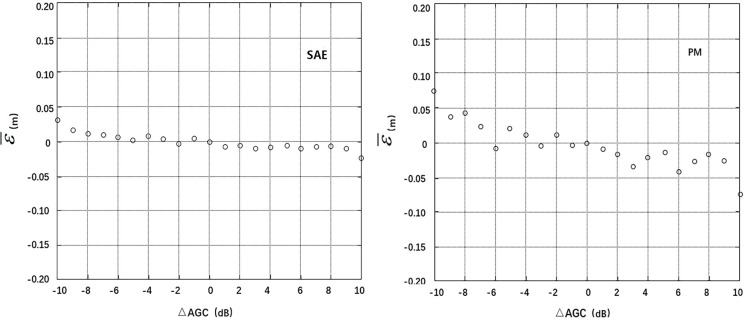
Distributions of mean residuals $\bar{\varepsilon }$ according to ΔAGC.

The deviations of SWH, U, σ_0_ and AGC at the cross points were divided into segments (0.5 m, 1.0 m/s, 1.0 dB and 1.0 dB), and the residual SSH error at each segment was averaged. Thus, the correlation between the mean residual error $\bar{\varepsilon }$ and the deviations **Δ**SWH, **Δ**U, **Δ**σ_0_ and **Δ**AGC can be analyzed. A smaller absolute value and standard deviation of the mean residual error $\bar{\varepsilon }$ at each segment indicates smaller correlations with **Δ**SWH, **Δ**U, **Δ**σ_0_ and **Δ**AGC are a more effective model [4]. The calculation results are listed in Table 1, and the residual error distribution maps are shown in Figs 4–7.

**Table 1:** Residual error analysis of the SAE model and the parametric model.

	SAE model (SAE)		Parametric model (PM)	
	Mean (cm)	Std (cm)	Mean (cm)	Std (cm)
$\bar{\varepsilon }$-_ΔSWH_	0.50	2.80	1.59	7.56
$\bar{\varepsilon }$-_ΔU_	0.48	1.78	1.31	2.87
$\bar{\varepsilon }$-_Δσ0_	0.55	3.70	1.30	7.55
$\bar{\varepsilon }$-_ΔAGC_	0.60	3.75	1.38	7.05

The statistical data in the table and the residual error distribution map in Figs 4–7 show that the residual error distributions of the SAE model for the four variables are closer to the zero line and exhibit smaller fluctuations than the residual error distributions of the parameter model. Thus, the SAE model is more effective for SSB estimation.

### Operational efficiency

Based on a computer running a Windows 10 operating system with a 3.6 GHz CPU, 32 GB of memory and running MATLAB R2017b, the execution times of the different models were recorded. The selected data sets were the 6 cycles of Jason1/2 (2,583,776 groups) and the 2 cycles of HY-2A (798,726 groups), and the models used are the SAE model, the parametric model and the nonparametric model. The execution times are listed in Table 2.

**Table 2:** Model execution times.

	SAE model (T_SAE_ /s)	Parametric model (T_PM_ /s)	Nonparametric model (T_NP_ /s)
6 cycles of JASON1/2 (2,583,776 groups)	871	560	10,800
2 cycles of HY-2A (798,726 groups)	305	190	3,600

As Table 2 shows, the calculation time of the SAE model is similar to that of the parametric model and is much faster than the nonparametric model; its efficiency is approximately 12 times higher than the nonparametric model. However, the SAE model accuracy is similar to that of the nonparametric model results because it was trained on the results of the nonparametric model.

## Conclusion

In this paper, a SAE model with 5 input parameters (SWH, U, SSH, σ_0_, AGC) was constructed and tested using Jason1/2 radar altimeter data. The model is composed of 3 hidden layers and the nodes of the hidden layers are arranged as 5, 10, 5.

The trained SAE model was validated by HY-2A radar altimeter data. A comparison analysis showed that the SSB computed by the SAE model is similar to the SSB of the parametric model in the GDR of HY-2A, although some differences exist. The results of the explained variance and residual error analysis showed that both the effectiveness and the SSB accuracy of the SAE model are better than the effectiveness and the SSB accuracy of the parametric model. In terms of operational efficiency, the execution time of the SAE model is similar to that of the parametric model and significantly better than the execution time of the nonparametric model.
